# Corporate Green Bonds: Understanding the Greenium in a Two-Factor Structural Model

**DOI:** 10.1007/s10640-021-00585-7

**Published:** 2021-08-02

**Authors:** Elettra Agliardi, Rossella Agliardi

**Affiliations:** 1 Department of Economics, University of Bologna, piazza Scaravilli 2, 40126 Bologna, Italy; 2 Department of Mathematics, University of Bologna, Bologna, Italy

**Keywords:** Green bonds, Climate finance, Corporate social responsibility

## Abstract

A novel structural model is developed to understand the determinants of green bond prices and the so-called ‘greenium’, that is, the premium that bondholders are willing to pay to invest in green securities rather than conventional ones. The presence of a greenium makes green bonds relatively cheap vehicles to fund environmentally sustainable projects and thus contributes to the shift to a green economy. Yet, evidence on the greenium is mixed and the determinants of green bond yields are not fully understood. In this model two sources of uncertainty are introduced, that is, of cash flows of the firm and of the effectiveness of the financed green projects. The adoption of two risk factors brings in some mathematical complexity but allows for a better modelling of the multi-facet nature of these financial instruments. Our model is rich enough to generate both a positive and a negative premium, as both have been detected in the empirical literature. Thus, we shed light on possible heterogeneity concerning the existence of a greenium in the green bond universe. Moreover, we show how green bonds affect the issuer’s creditworthiness, depending on the correlation of the green project with the core business of the firm and study their impact on investors’ portfolio allocation.

## Introduction

The peril of environmental damage and the need of moving financial investments towards green and environmentally sustainable projects have attracted both institutional and private investors in recent years. Although the priority today is to support overwhelming health systems and workers who are becoming unemployed due to Covid-19 crisis, thinking ahead, the response of the economy will continue offering tools to promote a green transition and build a more sustainable future.^1^

Since the first issuance in 2007 green bonds have emerged as a key financial tool to address the new challenges. Their distinguishing feature is that the proceeds are directed to projects with environmental benefits, primarily climate change mitigation and adaptation. The development of process guidelines (e.g. Green Bond Principles) promoting transparency and integrity in the green bond market has further encouraged the investment into green deals and increased their appeal to socially responsible investors.

Green bonds are the same as conventional bonds in every way except that the use of the proceeds is defined as green, and they are driven by the same financial risk factors that affect conventional bonds, therefore, in principle the reward to investors should be the same as from other bonds of the same issuer and with equal seniority. Sometimes green bonds exhibit a common new issuance premium in line with the standard bond market. At the same time, green bonds are usually oversubscribed. On one hand, additional costs related to ‘green’ certification and third-party review that are incurred by issuers should be reflected in bond prices. On the other hand, a higher degree of transparency—related to periodic monitoring and reporting in the post-issuance phase of the certification process—might result in a lower bond spread, as recognized for general corporate bonds (e.g. Yu 2005, where the transparency-related component of the bond spread has been identified; Clarkson et al. 2013; Plumlee et al. 2015, where it is shown that a higher quality environmental disclosure and externally produced environmental information are valued positively by investors). For green bonds, additional assessment is meant to protect investors from green-washing—that is, activities ‘disseminating a misleading picture of environmental friendliness’ (Bènabou and Tirole 2010)—and is often outsourced to an external auditing entity.

In this paper we provide an analytical framework for understanding the dynamics and the relevant risk factors of corporate green bonds. In particular, we study the complicated interactions among the various determinants of green bond prices, which are crucial to explain the so-called ‘greenium’, that is, the possible premium paid by bondholders for green bonds when compared with conventional bonds.

Although there is no unanimity on the subject, empirical analyses have often found evidence of a ‘greenium’, that is, a negative premium that makes green bonds more expensive to investors than other bonds from the same issuer. A negative premium is regarded favorably by issuers because it can lower their funding costs, while investors will receive a slightly lower yield compared to existing similar bonds. This negative premium to bond holders is not a straightforward outcome of the green labeling as the green certification is not a financial standard and does not imply any direct impact on the credit rating of a bond. On the other hand, an indirect credit quality enhancement might result from the transparent information flow related to the process of verifying the green credentials of a bond, which include alignment with the Green Bond Principles, mandatory requirements for use of proceeds, tracking, and reporting, and an assurance framework with independent verifiers and clear procedures.^2^

A common explanation for the greenium is that these instruments are appealing to investors with a green mandate, and socially responsible investors are an increasingly relevant liquidity source (Zerbib 2019).

On the whole, the literature on Corporate Social Responsibility (CSR) is not unanimous in relating responsible investing and reduction of financial risk (e.g., McWilliams and Siegel 2001; Amel-Zadeh 2018), and this applies also to most analyses of green investments.^3^ Most empirical studies investigate the different financial performance between ‘green’ and ‘brown’ companies mainly considering equity portfolios (see Alessi et al. 2021 and reference therein). Investors tastes and preferences can affect equity (Gollier and Pouget 2012; Gollier 2017), so that in addition to screening out undesirable stocks, investors might influence environmental policies of firms through shareholders proposals and lobbying the management. Some studies have tried to characterize the green mandate of those who are buying green bonds, but the takeaway of most analyses is that green bonds are being supported by those declaring themselves as green investors^4^ and those that do not (CBI 2018; Connaker and Madsbjerg 2019). Thus, green bonds apparently appeal to a wide range of investors which will be critical in scaling up the market. A theoretical model explaining why green assets exhibit lower expected returns has been developed in Pastor et al. (2020) in a CAPM framework where an environmental, social and governance (ESG) factor is introduced to capture customers’ tastes for green products and investors’ biases for green holdings. Their model can produce several predictions regarding green asset prices and portfolio holdings, the size of ESG investments and their impact at the social level. However, the assumption of normal returns and the restriction to market risk limits its application to corporate bonds, where credit risk is an important consideration.

Our focus is on corporate green bonds. Since the first corporate green bond issuance in 2013, the corporate green bond market has been expanding at an impressive rate. In 2019 non-financial corporates almost doubled issuance from 2018 representing 23% of the total volume (CBI 2020). The non-financial corporates almost reached the volume issuance of financial corporates, totalizing 205 bn $ in 2020.

There are very few studies focusing specifically on corporate green debt. Most studies have developed empirical analyses on a broader set of bond issuers, including sovereign and municipal bonds. In Sect. 2 we discuss some empirical evidence on green bond prices and conclude that even though results are mixed, a negative premium to investors prevails, albeit low on average.

To our knowledge, a theoretical explanation of the relationship between green and conventional bond prices is still an open question. In this paper a structural model is developed to explain the formation of green bond prices and to address the issue of the so called ‘greenium’. We model the basic mechanism behind green bond issuance by a corporation in a stylized way, but, at the same time, we try to address some complexities embedded in green projects. In particular, the issue of uncertainty related to green investments is modelled explicitly.^5^ While green bonds are characterized by a special type of communication with investors regarding the financed projects and their environmental impact, a precise assessment of their beneficial effects is difficult to elaborate especially because green projects often require new technologies, and reliable data on their effectiveness may be unavailable. This holds especially for those projects having long construction and operation periods such as those engaging in climate-change solutions. An additional source of uncertainty surrounding green projects may arise from the possibility of misuse of green funds and green-washing, i.e. when projects of ambiguous environmental value are financed, which shakes market confidence in these financial instruments.^6^ Our model, albeit stylized, is the first introducing both uncertainty about firm earnings and uncertainty about the effectiveness of the financed environmental projects, and their interplay. The contribution of our paper is as follows. First, our model is rich enough to generate both a negative and a positive premium, as both have been detected in the empirical literature, and provides an explanation for both occurrences. Second, we investigate how a green bond issuance affects an issuer’s creditworthiness, depending on whether the funded green project is correlated with the core business of the firm or is mainly undertaken to signal a philanthropic commitment. In particular, we find that although uncertainty reduces the firm’s advantage in committing itself to environmentally responsible projects in many cases, it may even become an opportunity if the green projects are correlated with the core business of the firm. Third, we model the demand side of the green bond market to provide an explanation for the attractiveness of such securities despite their relatively expensive price.

Thus, we complement the empirical investigation with a theoretical model that allows us to answer questions such as: How does the issuance of green bonds affect a firm’s ability to raise debt and its cost of financing? Does it make any difference across diverse issuers, i.e. ‘pure players’ whose revenues derive almost entirely from ‘green’ business activity vs firms with a large portion of revenue coming from another business line? What drives the corporate issuers’ supply and the investors’ demand for green bonds?

In Sect. 3 a structural model is introduced to explain the role of several determinants of the green bond price dynamics. An explicit solution is obtained and calibrated in Sect. 4. In Sect. 5, sensitivity analysis is performed with respect to the relevant parameters. Section 6 develops a model of optimal asset allocation for a portfolio of green vs conventional bonds and provides an explanation for the counterintuitive oversubscription of green bonds even in the presence of a negative premium to bondholders, and relate it to the investors’ environmental awareness. Finally, Sect. 7 concludes.

## Empirical Studies of Green Bond Prices

Despite the rapid development of a green bond market over the recent years, there is significant demand–supply mismatch, as the increased awareness of environmental issues pushes up the demand for green bonds. As a result, green bond issues tend to be oversubscribed and a common perception is spreading that green bonds pay lower yields than comparable conventional bonds without a green label. This alleged ‘negative’ premium to bondholders has been dubbed ‘greenium’ and has started being analysed in empirical studies. A report by the Climate Bonds Initiative is the first to address the question whether or not a green bond premium exists (CBI 2017). The conclusion does not provide clear evidence: they find that some green bonds are priced inside the issuer’s yield curve, some are priced on it, and others are priced above. Further studies reveal that some green bonds have generally been issued slightly above the yield curve and have continued to be priced in line with the issuers’ other bonds in the secondary market, although most of them considered only a limited number of bonds. The first academic study addressing the question of the existence of the greenium is Zerbib (2017), later published in a deeply revised version (Zerbib 2019), where a matching method is used to calculate the yield of an equivalent synthetic conventional bond for each green bond issued on December 30, 2016. The green premium is obtained as an unobserved specific effect of the regression of the difference in yields between the two bonds on the difference in liquidity. The conclusion is that there is a statistically significant negative premium on green bonds yield, which is quantified and explained on the basis of the bond characteristics. In particular, for Investment Grade bonds, the average premium is negative in various market segments such as EUR bonds (− 2 basis points), EUR bonds with a rating lower than AAA (− 4 basis points), USD bonds (− 5 basis points), and USD bonds with a rating lower than AAA (− 9 basis points). Using a different sample, Hachenberg and Schiereck (2018) do not find significant differences between yields of green bonds and conventional bonds of the same issuer on average, but some differences exist by rating class. Bachelet et al (2019) provide mixed results, suggesting for example that issue type (institutional vs private) and other characteristics (liquidity and volatility) matter for the sign of a premium.

Karpf and Mandel (2018) study the US municipal bond market and obtain an average positive premium of 7.8 bps for green bonds with rather mixed findings when the evolution over time of the greenium is analysed. Specifically, they find a higher yield for green bonds compared to the yield of conventional bonds in the period going from 2010 to 2014, whereas in the last 2 years (2015 and 2016) the yield of green bonds turns out to be lower than that of conventional bonds. Differently from Karpf and Mandel (2018), Baker et al. (2018) find that green bonds are priced at premium and earn on average 6bps lower returns than conventional bonds. They recognize that the different results may be due to the fact that they consider after-tax yields at issue, whereas Karpf and Mandel (2018) did not take into account the effect of taxes on yields. Since many green municipal bonds are taxable, higher yields at issuance are more likely (as found by Karpf and Mandel). However, if taxes are considered, yields for green bonds become lower than those of conventional bonds.

Barclays (2015) provides also other possible explanations for a negative premium suggesting that prices of green bonds reflect their externalities or a preference of investors (e.g. investors may gain additional benefits from green bonds) or a difference in risk and volatility.

Some studies find support for a greenium specifically in the corporate bond sector estimated between 6 and 24 bps (Gianfrate and Peri 2019; Tang and Zhang 2020) and the greenium seems to be more pronounced in later sample years (Lӧffler et al. 2021), and when there is an external certification of the bond greenness (Kapraun and Scheins 2019). In contrast, Kapraun and Scheins (2019) find that corporate green bonds may trade at a discount, particularly when they suffer from the worse valuation of the green label in the secondary market.

A recent analysis by Larcker and Watts (2020) provides perhaps the most critical study of the greenium by explicitly integrating a counterfactual for green bonds, however for US municipals only. They conclude that the premium due to “tastes” for green-labeling of bonds is basically zero.

A comparative analysis of the empirical literature on green bond prices is offered in Table 1.

**Table 1:** Literature review on green bond premium

	Bonds included in the study	Time period	Method	Primary/secondary market	Green premium
CBI (2016)	19 bonds	Q4 2016	Matching with iBoxx indices	Primary	Undecided
Barclays (2015)	Global Credit Index (investment grade) Global Green Bond Index—Securitized bonds are excluded	March 2014–August 2015	OLS regression	Secondary	Negative premium—17 bps
Ehlers et al. (2017)	21 green bonds compared to conventional bonds of the same issuers	2014–2017	Yield curve	Primary and secondary	Negative premium − 18 bps in primary market Similar performance of green indices in secondary market
Zerbib (2017, 2019)	110 GBP-aligned green bonds	July 18, 2013–Dec. 29, 2017	Control for liquidity	Secondary	Small but statistically significant negative premium
NN Investment Partners (2018)	133 labelled green bonds (Bloomberg MSCI Global Green Bond Index)	Dec. 2014–Nov. 2017	Yield curve	Secondary	Negative premium − 1.1 bps
CBI (2018)	60 green bonds	2016–2018	Yield curve	Primary	29 out of 60 green bonds have a negative premium
Karpf and Mandel (2018)	1880 green U.S. munis; 36,000 U.S. conventional bonds by the same issuers	2010–2016	Yield curve (Svensson 1994) Oaxaca- Blinder decomposition	Secondary	Positive premium + 7.8 bps
Baker et al. (2018)	2083 green U.S. munis 19 green U.S. corporate bonds	2010–2016 2014–2016	After tax-yield regressions	Primary and secondary	Negative premium − 5.7 bps (munis) Negative premium − 7.6 bps For CBI certified green bonds − 8.2 bps
Bachelet et al (2019)	89 bond couples	January 2013-December 2017	Matching pairs (amount, maturity, rating, coupon, currency, issuer)	Secondary	Positive premium for private non-certified green bonds, negative premium and higher liquidity for institutional issuers
Larcker and Watts (2020)	US munis, fixed rate coupon bonds 640 matched pairs	2016–2018 (overweighted later deals)	Matching pairs (like Crabbe and Turner, 1995)	Primary and secondary	The “greenium” is essentially zero
Hackenberg and Schiereck (2018)	63 bonds; 126 non-green bonds	Oct.1, 2015–March 31, 2016	Daily i-spreads Panel regression on size, rating, currency	Secondary	No significant premium
Gianfrate and Peri (2019)	121 green labelled (43 corporate issuers, 78 non-corporate issuers)	Jan. 2007–Dec. 2017	Propensity score matching and OLS regression of spreads (on variables used to estimate the PSM)	Primary and secondary	Significant greenium in EUR in primary market, more marked for corporations
Kapraun and Scheins (2019)	4500 matched bond pairs (1500 in primary market)	2009–2018	Regression on several variables	Primary and secondary	Negative, linked to green attributes in primary market; positive in secondary market
Tang and Zhang (2020)	1510 bonds (financial and industrial corporations, 132 public issuers)	Jun. 2007–Jul. 2017	Event study; regression of yield spreads on several variables	Primary	No significant greenium but benefits of green issuance to existing shareholders
Lӧffler et al. (2021)	1928 green bonds, 649 issuers	2007—2019	Coarsened exact matching	Primary and secondary	Time-varying significant greenium (more pronounced after 2018)

From a theoretical perspective, a difference in the valuation of green bonds from comparable conventional bonds can be partly understood in the case of sovereign or public entity’s issuances—where cash flows from green bonds are reinvested in green projects having government backing-, while a theoretical explanation of the greenium is not straightforward for corporate bonds, which convey an additional form of uncertainty in the realization of the green projects. Some literature suggests that investment in CSR—and thus in environmental projects—may reduce the sensitivity of the firm’s profitability to economic shocks (Orlitzky et al. 2003). Such resilience to negative shocks might also come from consumer loyalty by environmentally responsible consumers (Servaes and Tamayo 2013). It could also be a result of the Fama and French (2007) effect, that demonstrates that when a group of investors have a ‘taste’ for a certain type of assets (i.e., because of a pro-environmental motive) equilibrium prices shift and there may be implications on asset portfolios.

In any case, since the first corporate green bond was issued in 2013, the market for such securities has started evolving and its impressive expansion poses new challenges to the theoretical understanding of its mechanism.

In what follows, we focus on the case of corporate bond and present a structural model that is able to generate and quantify both a negative and a positive premium. Additionally, it offers a novel and comprehensive framework to understand the determinants of green bond prices and their interactions.

## Basic Model

The purpose of this section is to derive the green bond price expression, and thus the yield, to study the so-called greenium. Consider a firm with assets-in-place that generate uncertain earnings before interest and tax described by a stochastic process of the form:

1 $$dY_{t} = \mu Y_{t} dt + \sigma Y_{t} dW_{t}$$

where $W_{t}$ is a standard Wiener process with respect to an assigned filtration $\left\{ {I_{t} } \right\}_{t \ge 0}$. The size of $Y_{t}$ is determined by the amount of the firm’s output and this random variable is affected by the demand shock for the firm’s product as well.

Let us denote the market risk-free interest rate by *r* and the asset growth rate by $\mu$. The production process generates environmental damage, either because it employs polluting technologies or because the outputs do not fully conform to clean standards.^7^

Let *D* denote the damage per unit of production. We suppose that the firm income is reduced due to the internalization of damages through environmental policy. Let *p* denote the proportional reduction of the firm income. Our assumption is consistent with some evidence, e.g., Karpoff et al. (2005) who found that around announcement of the penalty for environmental regulation violations, the market value of the violating firms on average decreases by the size of the penalty. The firm may decide to exercise an investment option to contribute to an improved environmental quality via its activities. The list of the projects eligible for funding through green bond issuance shall conform to precise criteria and the resulting environmental benefits need to be shown in the subsequent Sustainability Reports. Let g > 0 denote the intensity of the investment expenditure in the green technology and let $\delta$ be a positive parameter representing the effectiveness of the mitigation policy. We suppose that the green investment allows to reduce the initial damage, $D_{0}$, by a factor $e^{ - \delta g}$. This factor might be replaced by any other decreasing function of *g*. To represent the uncertainty about the effectiveness of the newly adopted technology and/or the assessment errors regarding the beneficial impact from the green projects, we introduce a stochastic process, $X_{t}$, capturing the responsiveness to climate risk or any other forms of environmental risk. This additional source of uncertainty plays a key role in defying the purpose of green investments by hampering the efforts of a low-carbon transition or other environmental-friendly technologies. It also extends Agliardi and Agliardi (2019) by introducing a two-factor structural model, which permits to explain the new empirical evidence of both positive and negative greenium. While in Agliardi and Agliardi (2019) they adopt a traditional one-factor model for firm earnings and obtain the greenium effect in all circumstances, as market expanded and more empirical research on corporate green bonds became available, heterogeneous evidence on the greenium appeared (see Table 1). Therefore, a more comprehensive model is needed to capture the variety of empirical outcomes. This can be achieved introducing an additional risk factor as we do in this paper. Here we adopt a parsimonious model where the randomness is modelled through a stochastic process fluctuating around 1, that is, $X_{t}$ = $1 + s\tilde{W}_{t}$, with *s* > 0 and $\tilde{W}_{t}$ a Wiener process such that $E\left[ {dW_{t} d\tilde{W}_{t} } \right] = \rho dt.$

After engaging in the green investment net earnings are penalised by $pD_{g} X_{t} Y_{t}$, where $D_{g} = e^{ - \delta g} D_{0}$.

Note that the interpretation of this term as a penalty requires that $X_{t}$ preserves a positive sign over time, which would be guaranteed by a geometric (rather than an arithmetic) Brownian motion. However, in the sequel, *s* will be a small number, so the two stochastic processes can be (almost) safely confounded and the discussion below will be confined to positive values of *X*, which cover most situations occurring in practice. Thus, we assume that such penalty acts with a negative sign on Y, so that for negative (positive) values of the correlation parameter, $\rho$, the firm earnings and the environmental risk factor tend to move in the same (opposite) direction, that is, there is a positive (negative) correlation between the core business of the firm and the green technology funded by the issued green bond.

Although the exercise of the green option is irreversible, the firm has the flexibility to exercise the option at any time. In this model we assume that the firm funds its green policy by issuing a perpetual green bond with a continuous coupon. This assumption on bond life is not restrictive because, as Zerbib (2019) found, ‘the green bond premium does not appear to be significantly impacted by the maturity of the bond’ (p. 48).

In our model we suppose that the management chooses the exercise policy of the green option in order to maximize the market value of equity. Additionally, equity holders have the option to stop operations and trigger default in an optimal way. Let $Y_{I}$ denote the level of the fundamental variable Y at which it is optimal to issue a green bond, and let Y* denote the default threshold, i.e. default is triggered whenever Y falls below Y*.

The following requirements should be accomplished in order to model a green bond:

(i) all the proceeds from the bond issuance should be directed to funding green projects;
(ii) special reporting, monitoring and accounting are requested for attributing the green labelling to the bond.

We model (i) by assuming that the investment amount equals the bond value at the investment threshold, and we add a fixed cost, *K*, to represent the extra costs related to the requirements (ii).

In what follows, $\tau$ denotes the corporate tax rate and *g* denotes the contractual continuous coupon of the green bond. The coupon payment is tax deductible at the corporate tax rate.

Our objective is to derive the green bond price expression. Thus, we first compute the equity value which determines the default threshold, Y*, and the optimal level of *Y* for green bond issuance, $Y_{I}$. The default trigger Y* is endogenously and optimally chosen by equity holders by maximizing equity value. When earnings drop to Y*, then the firm goes bankrupt and debt holders take over and obtain the firm’s unlevered assets net of bankruptcy costs. On the other hand, if earnings rise to $Y_{I}$, then the firm makes the green project investment financed by green bonds. The optimal timing for investment, and thus for issuing green bonds, is chosen to maximize the market value of equity. Once $Y_{I} {\text{}}$ and Y* are determined, we can compute the debt holders’ claims.

Following usual arguments in corporate finance, the post-investment equity value, *V(X,Y)*, is found by maximizing the expectation of the discounted cash flows from the net earnings, that is:

2 $$V\left( {X,Y} \right) = sup_{t*} E[\mathop \smallint \limits_{0}^{t*} e^{ - rt} [\left( {Y_{t} - g} \right)\left( {1 - \tau } \right) - pD_{g} X_{t} Y_{t} \left] {dt} \right]$$

where t* is a stopping time with respect to the filtration $\left\{ {I_{t} } \right\}_{t \ge 0}$ and $X_{0}$ = X, $Y_{0}$ = Y. Here t* represents the default time, i.e., the first time that equity value drops to zero. The boundary-value formulation of this problem is:

3 $$\Psi V + \left[ {Y - g} \right]\left( {1 - \tau } \right) - pD_{g} XY = 0$$

with the boundary conditions:

4 $$V\left( {X,Y*\left( X \right)} \right) = 0,\quad \partial_{Y} V\left( {X,Y*\left( X \right)} \right) = 0,\quad \partial_{X} V\left( {X,Y*\left( X \right)} \right) = 0,\quad \lim_{Y \to \infty } V\left( {X,Y} \right)/Y = < \infty \; {\text{for}}\,{\text{ any}}\;{\text{X}} > 0$$

and the differential operator $\Psi$ is defined as follows:

5 $$\Psi = \frac{{\sigma^{2} }}{2}Y^{2} \partial_{Y}^{2} + \mu Y\partial_{Y} - r + \frac{{s^{2} }}{2}\partial_{X}^{2} + \rho \sigma sY\partial_{XY}^{2}$$

Here Y*(*X*) represents the boundary between the continuation region and the stopping region where the firm stops operations by triggering default. The first three conditions in (4) are the smooth-pasting conditions needed for optimality of the decision to default, while $\lim_{Y \to \infty } V\left( {X,Y} \right)/Y < \infty$ is the no-bubble condition for equity value.

A particular solution of (3) is found in the form:

6 $$Vp\left( {X,Y} \right) = Z_{g} Y - \frac{{g\left( {1 - \tau } \right)}}{r} - P_{g} XY$$

where $$P{}_{g}$$ = $$\frac{{pD_{g} }}{r - \mu }$$ and $$Z_{g}$$ = $$\frac{{1 - \tau - \rho \sigma sP_{g} }}{r - \mu }$$.

Then we look for a solution of the homogeneous equation of the form $A\left( X \right)Y^{\beta }$ and write a general solution of (3) as $A\left( X \right)Y^{\beta }$ + *Vp(X,Y),* along the lines of the method used by Adkins and Paxson (2011) for two-factor stochastic models. Unfortunately, this approach only allows to obtain an approximate solution and this kind of difficulty is shared by most problems with two-factors uncertainty. In the “Appendix” we provide the details of this solution method, which is legitimate in a suitable neighbourhood of X = 1, while the error cannot be neglected when X differs significantly from its anchor value.

The boundary conditions yield:

7 $${\text{Y}}* \, = \frac{\beta }{\beta - 1}\frac{{g\left( {1 - \tau } \right)}}{{r\left[ {\Omega \left( X \right)} \right]}}\quad {\text{and }}A(X)=\quad \frac{1}{- \beta }\left[ {\frac{\beta }{\beta - 1}\frac{{g\left( {1 - \tau } \right)}}{r}} \right]^{1 - \beta } \Omega (X)^{\beta }$$

where

$$\Omega \left( X \right) = Z_{g} - P_{g} X,$$

and

$$\beta = \left[ {\frac{{\sigma^{2} + (sz)^{2} }}{2} - \mu - \sqrt {\left( {\frac{{\sigma^{2} + (sz)^{2} }}{2} - \mu } \right)^{2} + 2r(\sigma^{2} + (sz)^{2} - 2\rho \sigma sz}) } \right]\left / \right[\sigma^{2} + \left( {sz)^{2} - 2\rho \sigma sz} \right]$$

with $$z = P_{g} /\Omega \left( 1 \right)$$.

We assume that the parameter values and X are such that Ω(X) > 0, so that Y* > 0, that is, default is a possible event.

In the special case *s* = 0 (deterministic damage), we get an exact solution with

8 $$\beta = \frac{1}{2} - \frac{\mu }{{\sigma^{2} }} - \sqrt {\left( {\frac{1}{2} - \frac{\mu }{{\sigma^{2} }}} \right)^{2} + \frac{2r}{{\sigma^{2} }}} \quad {\text{and}}\quad Y^{*} = \frac{\beta }{\beta - 1}\frac{{g\left( {1 - \tau } \right)\left( {1 - \mu /r} \right)}}{{\left( {1 - \tau - P_{g} \left( {r - \mu } \right)} \right)}}$$

In this case, the equity value takes the form

9 $$V\left( Y \right) = \left[ {\frac{1 - \tau }{{r - \mu }} - P_{g} } \right]Y - \frac{{g\left( {1 - \tau } \right)}}{r} + \frac{{g\left( {1 - \tau } \right)}}{{r\left( {1 - \beta } \right)}}\left( {\frac{Y}{Y*}} \right)^{\beta }$$

Overall, in the general case and with negative correlation values, the effect is similar to an enhancement of the volatility which results in an increase of the equity value. Moreover, this effect is positively correlated with the risk parameters, *s* and $\sigma$, and with the absolute value of the correlation parameter $\rho$.

In the sequel we consider an approximate solution where $\Omega \left( X \right)$ is replaced by $\Omega \left( 1 \right)$ and for notational convenience the corresponding value of $\beta$ will be denoted by $\overline{\beta }$. This amount to solving the two-factor model ruling out too risky green projects.

Let $Y_{I}$ (X) denote the level of Y at which it is optimal to issue a green bond and to invest in the green technology. We suppose that equity holders choose the investment threshold $Y_{I}$ to maximize the ex ante equity value. Let us denote $Y_{I}$ (1) by $Y_{I}$ to simplify the notation. The ex ante equity value is of the form: $\left[ {\frac{1 - \tau }{{r - \mu }} - P_{0} } \right]Y + \hat{A}Y^{{\beta_{ + } }}$, with $\beta_{ + } > 0$, where the latter term $\hat{A}Y^{{\beta_{ + } }}$ represents the investment option. Here $\beta_{ + }$ is the positive solution of:

$$\frac{{\sigma^{2} }}{2}\beta \left( {\beta - 1} \right) + \mu \beta - r = 0.$$

By matching the ex ante and ex post values and their derivatives at the point $Y_{I}$, the investment threshold and the arbitrary constant $\hat{A}$ can be determined. We add up an extra cost, *K*, to the ex ante firm value, which embodies the additional expenditures related to a green bond issuance (extra investment costs, separate accounting, additional monitoring and reporting, etc.). In particular, $Y_{I}$ is obtained solving the following equation:

10 $$\frac{{g\left( {1 - \tau } \right)}}{r}\left\{ {\frac{{\beta_{ + } - \overline{\beta }}}{{\overline{\beta } - 1}}\left( {\frac{{Y_{I} }}{Y*}} \right)} \right.^{{\overline{\beta }}} + \left. {\beta_{ + } } \right\} + \left( {1 - \beta_{ + } } \right)\left[ {P_{0} - P_{g} \left( {1 + \frac{\rho s\sigma }{{r - \mu }}} \right)} \right]Y_{I} + \beta_{ + } K = 0$$

In what follows, we restrict ourselves to those situations such that $Y_{I} > Y^{*}$.

Having computed $Y^{*} ,$ we can turn to computing the current value, *G*, of the green bond. The bond is issued at the investment time $Y_{I}$. If the level of the fundamental variable Y is above the default threshold Y*, then *G(Y)* satisfies the following equation:

11 $$\Psi G + g = 0$$

where $\Psi$ has been defined in (5) and *g* is the contractual continuous coupon. A trivial solution of (11) is *G(Y)* = $\frac{ g}{r}$, which represents the value of a default-free bond. We need to find a solution matching the recovery claim at default. Upon default bondholders receive the firm value (evaluated at Y*) minus bankruptcy costs amounting to the fraction α (0 ≤ α ≤ 1) of firm value. Let us write the bond value in the form:

12 $$G\left( {X,Y} \right) = \frac{g}{r} + \hat{G}\left( {Y^{*} \left( X \right),Y} \right)$$

where $\hat{G}\left( {Y^{*} ,Y^{*} } \right) = \left( {1 - \alpha } \right)\left[ {\frac{1 - \tau }{{r - \mu }} - P_{g} } \right]Y^{*} - \frac{g}{r}$, and the first term of $\hat{G}\left( {Y^{*} ,Y^{*} } \right)$ represents the value of the recovery claim at default.

By using the same approximation as above to solve Eq. (11), we write $\hat{G}$ in the form:

13 $$\left( {1 - \alpha } \right)\left[ {\frac{1 - \tau }{{r - \mu }} - P_{g} } \right]Y^{*} \left( {\frac{Y}{{Y^{*} }}} \right)^{{\beta_{1} }} - \frac{g}{r}\left( {\frac{Y}{{Y^{*} }}} \right)^{{\beta_{2} }}$$

where

$$\begin{aligned} & \beta_{1} = \frac{1}{2} - \frac{\mu }{{\Sigma_{ - }^{2} }} - \sqrt {\left( {\frac{1}{2} - \frac{\mu }{{\Sigma_{ - }^{2} }}} \right)^{2} + \frac{{2(r - \left( {sz)^{2} } \right)}}{{\Sigma_{ - }^{2} }}} , \\ & \beta_{2} = \frac{1}{2} - \frac{\mu - \rho \sigma sz}{{\Sigma_{ - }^{2} }} - \sqrt {\left( {\frac{1}{2} - \frac{\mu - \rho \sigma sz}{{\Sigma_{ - }^{2} }}} \right)^{2} + \frac{2r}{{\Sigma_{ - }^{2} }}} , \\ & \Sigma_{ - }^{2} = \sigma^{2} + (sz)^{2} - 2\rho \sigma sz. \\ \end{aligned}$$

Expression (12)–(13) is the green bond price which will be calibrated in Sect. 4 and discussed in Sect. 5.

## Calibration of the Model

In this section we calibrate our model by both adopting the existing literature parameters and matching some parameter values to empirical data. We apply our model to a case study, that is, the green bond issued by Hera, identified by the ISIN Code XS1084043451. Hera is a multi-utility group with a broad business portfolio and was the first to launch this financial security in Italy. The sample size consists of 1007 observations of bond daily prices for the period between July 4, 2014, and May 14, 2018. This green bond (see Table 2) exhibits a lower bid-ask spread than conventional bonds which is related to a higher liquidity. At issuance, the bond was very successful, with subscriptions for a notional value of about three times the amount of the bond. Table 2 presents the descriptive statistics for the spread. The mean value and standard deviation of the MAE (mean absolute error) are 5.31 bps and 3.53 bps, respectively, and the average negative premium is of 83.6 bps. The “greenium” effect is clearly shown and its size is above the average values—however included in the range of values—found in the empirical literature on corporate green bonds.

**Table 2:** The ‘greenium’ for Hera bond

ISIN code	XS1084043451	Issue type	Fixed rate	Listing date	04/07/2014
Issued amount	500 000 000 EUR	Coupon	2.375%	Final maturity	04/07/2024

	Negative spread		Negative spread		
Mean	83.6 bps	First quantile	68.3 bps		
Max	133.7 bps	Second quantile	81.1 bps		
Min	0 bps	Third quantile	91.6 bps		

We implement our model by using the following parameter values: *r* = 2.1% is extracted from the EU yield curve at the time of observation, μ = -0.83% and σ = 23.86% are estimated from the stock returns in the years preceding the bond issuance. The Italian corporate tax rate is 27.9%, while bankruptcy costs are assumed at the 15% level, in keeping with empirical literature in corporate finance (e.g. Altman and Hotchkiss 2006). As the number of green projects funded throughout this Hera bond is pretty large (25 in 2014) and their nature is diverse (i.e., energy production by non-fossil fuel, fight against climate change, increase of energy efficiency, air quality, improvement of wastewater treatment plants, clean water, increase of sorted waste collection and disposal and reduction of waste disposal in landfills) the overall environmental benefit is hard to estimate. Thus, we perform a rough calibration of the ‘environmental’ parameters in order to achieve a perspective reduction of 50% of the environmental damage. Given the above parameters, we evaluate the yields and thus the greenium. If we neglect the environmental uncertainty we get a greenium of 37 bps at issuance, which is significantly lower than the average empirical greenium. This result can be improved if we assume a non-vanishing *s* (e.g. *s* = 5%) and a negative value for the correlation parameter $\rho$, representing a positive correlation between the green project and the firm business lines: for example, we get a greenium of 55 bps with $\rho$ = -0.5 or 65 bps with $\rho$ = -0.8. As usual, a classical structural model generates lower theoretical values for the credit spreads. This drawback could be removed by adopting an incomplete information framework (see, e.g., Giesecke and Goldberg 2004), but it would introduce mathematical complication and is beyond the scope of this work.

In the next section, sensitivity analysis is performed on a base case and the implications of the model assumptions are discussed in detail.

## Sensitivity Analysis and the ‘Greenium’ Effect

In what follows, we perform numerical computation to compare the green bond value and yield at issuance to an otherwise comparable non green, or conventional, bond. The expression of the conventional bond can be obtained from (12), taking $D_{g} = D_{0} {\text{ and }}s = 0.$ Our aim is to clarify the role of the several model parameters and, in particular, how they interact in determining a positive or negative premium.

Let us fix the following parameter values which are close to those of our case study: *r* = 4%, μ = − 1%, σ = 20%, for the risk-free interest rate and the firm relevant variables, respectively, while *s* = 10%, $\rho = - 0.5$, p = 0.1, δ = 0.5 and *K* = 0.01. Bankruptcy costs are assumed at the 15% level, as usual in the empirical literature in corporate finance, while the corporate tax rate is 30%. The current value of a related risk-free bond is fixed at 100.

In the simulations the greenium is defined as the difference between the yield on a conventional bond and the yield on a comparable green bond, so that a positive value illustrates the so-called greenium effect. By comparing the yield on the green bond and on an equivalent conventional bond, we generally obtain a negative spread from the perspective of green bondholders: for example, the greenium at issuance is 12.36 bps. With these parameter values the default threshold is reduced by about 26% if the conventional bond is replaced by a green bond, because engaging in the green investments is beneficial to the firm earnings, which ultimately leads to an indirect enhancement of the firm’s credit quality.

**Fig. 1:**
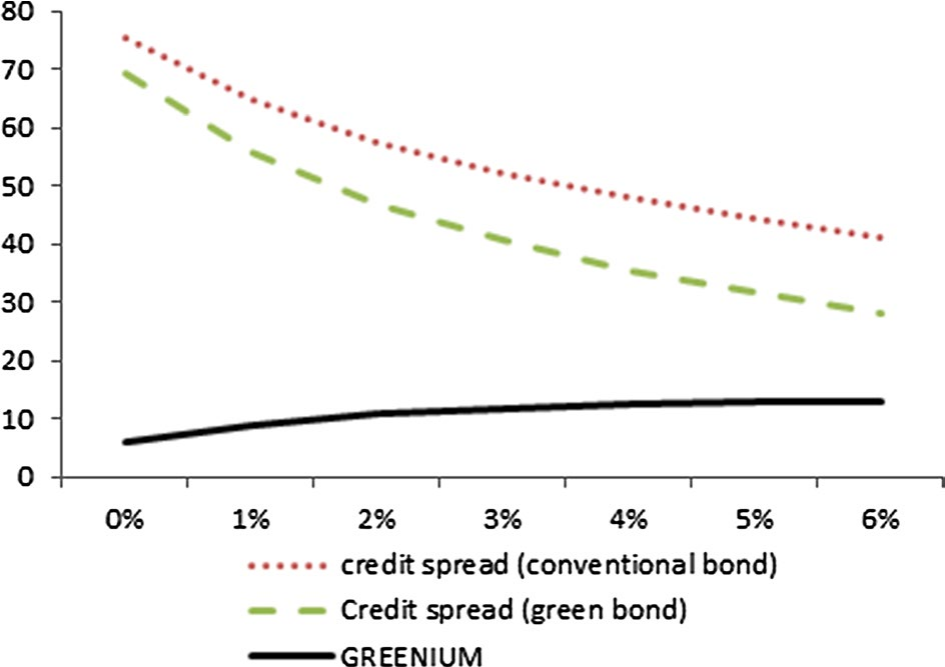
Credit spread and greenium (in bps) as a function of r

Figure 1 illustrates the sensitivity of the greenium to the risk-free interest rate, *r*, where all other parameters are as in the base case. While the credit spread in the primary market decreases for both bonds when r increases—as expected—, the greenium tightens when interest rates are lower (in our plot the greenium ranges from about 6–13 bps).

This may, at least partly, explain why a less pronounced greenium effect has been detected in the recent times of low interest rates worldwide. As interest rates tend to rise green bond might come with a higher greenium. Of course this effect is dampened by liquidity effects that work in the opposite direction.

It is interesting to analyse the impact of the uncertainty related to the effectiveness of the green investment. In Table 3, the uncertainty parameter of the green projects, *s*, is varied while all other parameters are left unchanged and two different values for the correlation parameter are used. Intuitively, uncertainty in the green projects is generally expected to reduce the competitive advantage of a firm committing itself to environmentally responsible investments, as is the case in the first row of Table 3, where the greenium decreases, but uncertainty may even become an opportunity whenever the green project has a positive impact on the usual firm earnings (see the second row in Table 3, where the greenium increases).

**Table 3:** Sensitivity of the greenium (bps) to the riskiness of the green projects (s) and correlation parameter ($\rho )$

s =	5%	10%	15%
$\rho = 0.5$	10.34	9.69	9.06
$\rho = -0.5$	11.67	12.36	13.06

On the other hand, performing sensitivity analysis with respect to the correlation between the asset volatility and the stochastic process governing the green technologies one gets a significant change in the greenium (see Table 4, where *s* is fixed at 50% while other parameters are as in the base case above). In some extreme situations the sign in the green premium is even reversed (e.g. Table 4 shows a negative greenium if $\rho$ = 1). Note that negative values of the correlation parameter, $\rho$, represent situations where the firm business gains a direct benefit from the adoption of a green technology, whereas positive values of $\rho$ model the case where the green project has no immediate positive impact on the corporate earnings—while introducing additional costs—, and the socially responsible corporate policy is adopted as a ‘delegated exercise of pro-social behaviour’ (Bènabou and Tirole 2010) or insider-initiated philanthropy. Even in this latter situation, where the immediate benefits are less well defined, an indirect positive return might result, for example, in the form of advertising tool and reputational enhancement. A deeper discussion on the understanding of Corporate Social Responsibility adoption is offered in Bènabou and Tirole (2010) and the related literature. Here we just emphasize that, overall, a corporate benefit in the form of a lower cost of capital is obtained in most situations through the issuance of green bonds.

**Table 4:** Sensitivity of the greenium (bps) to the correlation parameter ($\rho$)

$\rho =$	− 0.5	0	0.5	1
Greenium (bps)	18.3	11.3	5.0	− 0.3

As statistical investigation has started identifying different levels of issuers in terms of relative size of the ‘green’ business line,^8^ our finding might fuel empirical research on the precise impact of green bond issuance across several types of issuers. Unfortunately for many industry sectors insufficient reporting information is available to disentangle the share of ‘green’ revenues.

If we perform the analysis for the parameters governing the green technology (*p* and $\delta$) we obtain that an improvement related to a more effective technology widens the greenium, as expected.

Finally, a decrease in the corporate tax rate reduces the tax benefit of the debt service and shrinks the negative spread to the green bond holders (see Table 5, where all parameters are as in the base case except the corporate tax rate).

**Table 5:** Greenium (bps) versus corporate tax rate ($\tau$)

$\tau =$	10%	20%	30%	40%
Greenium (bps)	5.96	8.37	12.36	19.65

## Modelling the Bondholders’ Side

In this section we study the circumstances under which bondholders prefer green bonds to other forms of investments. Specifically, we model a problem of optimal portfolio allocation between a green bond and a money-market account, and a similar problem for a conventional bond. We consider a prototypical (representative) consumer-investor who faces the problem of how to optimally split his wealth between consumption and investment, and employs an Epstein-Zin utility function. The choice of such function is motivated by an abundant literature in environmental economics (see, e.g., van der Ploeg 1993; Ackerman et al. 2013; Traeger 2014) claiming that it is the most appropriate framework for optimization problems as it allows for disentangling the risk aversion attitude from the elasticity of the intertemporal substitution.

Consider the case of a consumer-investor who builds up a portfolio consisting of $N_{g}$ green bonds while the remaining part of his wealth that is not consumed earns at a riskless rate, *r*. Let *c(t)* denote the consumption rate and let *R(t)* denote the total wealth at time *t*. Let $w = \frac{{N_{g} G}}{R}$ denote the share of wealth invested in the green bond, where the current bond value, *G*, has been computed in Sect. 3 in terms of the two risk factors. Then the consumer-investor’s budget equation is:

14 $$dR = wR\left[ {\frac{dG}{G} - rdt} \right] + rRdt - c\left( t \right)dt$$

In view of the expressions (12) and (13) for the green bond value, *G*, and restricting our argument to the case $Y > > Y^{*}$, that is, default is a remote occurrence given the current level of the firm fundamentals,^9^ we can simplify the expression for *dG* as follows:

$$\frac{dG}{G} = \frac{{z\beta_{2} }}{r}\left( {\hat{g} - r} \right)dX + \frac{{\beta_{2} }}{r}\left( {r - \hat{g}} \right)\frac{dY}{Y}$$

where $\hat{g}$ = g/*G* is the yield on the green bond. Thus, the budget equation can be rewritten as follows:

15 $$dR = wR\left[ {\frac{{zs\beta_{2} }}{r}\left( {\hat{g} - r} \right)d\tilde{W}_{t} + \frac{{\beta_{2} }}{r}\left( {r - \hat{g}} \right)\left( {\mu dt + \sigma dW_{t} } \right) - rdt}\right] + (rR - c)dt$$

Let us adopt a utility function of Epstein-Zin type of the form:

16 $$U\left( {c,J} \right) = \frac{\nu }{\varepsilon \xi }J((c( {\frac{J}{\xi })^{ - \xi } )^{\varepsilon } - 1})$$

where *J* is the value function of the optimal consumption-investment problem, $\nu$ is a discount parameter, $1 - \frac{1}{\xi }: = \gamma$ is the relative risk aversion parameter and $\frac{1}{1 - \varepsilon }$ represents the attitude toward intertemporal substitution (EIS). Typically, $\gamma$ > 1 and thus $\xi$ < 0.

The consumer-investor’s problem takes the form:

17 $$max_{w,c} \;{\text{E}}\left[ {\mathop \smallint \limits_{0}^{\infty } U\left( {c_{t} ,J_{t} } \right)dt} \right]$$

with the wealth process satisfying the budget Eq. (14) and the initial wealth, *Ro*, is given.

To simplify the notation let us denote:

$$a = \frac{{\beta_{2} }}{r}\left( {r - \hat{g}} \right)\quad {\text{and}}\quad \Lambda^{2} = (a\sigma )^{2} + (azs)^{2} - 2\rho a^{2} z\sigma s.$$

Then the Bellman equation is written as:

18 $$\max_{w,c} \left\{ {\frac{{\nu c^{\varepsilon } [J\left( R \right)/\xi ]^{1 - \varepsilon \xi } }}{\varepsilon } - \frac{\nu }{\varepsilon \xi }J\left( R \right) + \left[ {\left( {aw\mu + r\left( {1 - w} \right)} \right)R - c} \right]J^{{\prime }} \left( R \right) + \frac{{\Lambda^{2} w^{2} R}}{2}^{2} J^{{\prime \prime }} \left( R \right)} \right\} = 0$$

The first order conditions yield:

19 $$c = \left[ {\frac{{J^{{\prime }} }}{\nu }\left( {\frac{J}{\xi }} \right)^{\varepsilon \xi - 1} } \right]^{{1/\left( {\varepsilon - 1} \right)}} \quad {\text{and}}\quad w = \frac{r - a\mu }{{\Lambda^{2} }}\frac{{J^{{\prime }} }}{{RJ^{{\prime \prime }} }}$$

Let us search for a solution of the form: J(R) = $HR^{h}$ where *H* and *h* are to be determined. Plugging (19) into the Bellman equation we obtain an explicit expression for *H* and *h*, in particular, *h* = $1/\xi = 1 - \gamma$. Then we conclude that

$$w = \frac{a\mu - r}{{\gamma \Lambda^{2} }}$$

A similar computation can be performed by replacing the green bond with a conventional bond. In this case, the weights of bond investment in the portfolio, $\hat{w}$, turns out to be:

$\hat{w} = \frac{{\hat{a}\mu - r}}{{\gamma \sigma^{2} }}$ where $\hat{a} = \frac{{\beta_{ - } }}{r}\left( {r - \hat{b}} \right)$ and $\hat{b}$ is the yield on the bond.

Note that the share of green bonds is larger than the share of conventional bonds whenever

20 $$a\mu - r > \left( {\hat{a}\mu - r} \right)(\Lambda /\sigma )^{2} .$$

In general, this relationship is not satisfied if the greenium effect is present. Nevertheless, if we consider an investor whose utility function is affected by the degree of sustainability of consumption goods, then the portfolio allocation might be altered consequently. Fama and French (2007) showed that there may be price effects of asset “tastes”, for assets that are treated as consumption goods and investors have a “taste” for such assets (as is the case for socially and environmentally responsible investing). The introduction of green biases in the utility function of socially responsible agents has been adopted also in other studies (e.g. Pastor et al. 2020), where, however, the consumer-investor’s problem is not addressed. A parsimonious way to address this situation in our framework is to assume that, in the above-mentioned utility function, the consumption rate decreases with damage, so that consumers show preferences for environmental quality. To fix ideas, we replace *c* with *c*^*1*+*f(D)*^ where *f* increases in the damage, *D*, and *f(D)* ≥ *0.* As *f(D)* becomes very large the consumption rate shrinks to zero, due to the consumer’s environmental concern. Then the argument above gives the following bond allocation:

21 $$w = \, (a\mu - r)/[\Lambda^{{2}} \left( {f(D)\left( {\gamma - 1} \right) + \gamma } \right)]$$

In this case, inequality (20) may be reversed, especially when *f(D)* is large. In other words, the yield appetite on the demand side is mitigated by the investors’ green aspirations. Note that this effect acts in the same direction as γ. This links our argument to the issue of how social responsibility is related to the risk aversion attitude. As Andries (2008) notes: ‘Socially responsible investors would simply be the ones with higher risk aversion to a deterioration of the state of the world’.

We conclude that the worth consumers-investors attribute to environmentally sustainable products ultimately leads to an increased demand for green bonds and to issue oversubscriptions; this in turn provides a cheaper way to fund environmentally sustainable projects. This remark is in keeping with empirical literature documenting that the volume of green bonds allocated to investors who identify themselves as environmentally responsible is increasing and is expected to increase for emerging markets as well. Further evidence will be provided when the disclosure of investors’ preferences toward environmental sustainability will be fully integrated into the suitability obligations arising from EU Directive 2014/65 (MiFID II) and EU Directive 2016/97 (IDD).

## Conclusion

Despite the success of green bonds and the rapid evolution of the green bond market, the theoretical literature on these financial instruments is still scarse. Our paper provides an analytical framework for understanding the dynamics and the relevant determinants of corporate green bonds. We use a two-factor structural model for corporate bond valuation which allows us to disentangle two sources of uncertainty related to firm earnings and the effectiveness of the financed green project. A special focus is on the formation of the so-called greenium, that is, the common wisdom that green bonds price inside their own yield curve. As reported in Sect. 2, a significant evidence of this phenomenon has been documented, albeit the empirical literature is not unanimous in confirming this effect. Our numerical simulations mainly refer to the primary market where the greenium is more evident. However, our model is able to generate both a positive and a negative green premium, depending on the complicated interactions among the various determinants of green bond prices. In particular, we find that the size of the greenium is positively affected by more volatile asset prices, larger interest rate and corporate taxes, and, more importantly, we show that issuers’ creditworthiness depends on the correlation of the green project with the core business of the firm. If the firm core business gains a direct benefit from the adoption of the green project, then the greenium is larger than when the green policy is mainly implemented as a delegated exercise of pro-social behaviour, in which case the greenium may even reverse its sign. This result is important because it gives an indication on the benefits in the form of a lower cost of capital that firms in different sectors may obtain from issuing green bonds. Our result is consistent with Gianfrate and Peri (2019), who found that, in the primary market, the greenium is more pronounced for corporate issuers in the utility and power sector, while it is smaller for issuers whose core business is not strictly related with the green project. In the latter case, the greenium may even reverse in the secondary market. Our result opens the way to further empirical research on the impact of green bonds issuance across several types of issuers and sectors. Moreover, some further refinements of our model could be introduced, for example, some performance considerations in terms of book cover and other liquidity issues in the secondary market.

Another result of our paper is derived in Sect. 6, where we extend the model to study the impact on investors’ decisions from the perspective of asset allocation. Using a modified Epstein-Zin utility function, we study the effect of investors’ non-pecuniary preferences for environmental quality on portfolio allocation and show that the introduction of green biases in the utility function may revert the preference in favour of bonds that are less remunerative but more environmental-friendly.

There is statistical evidence that green bonds achieve larger average oversubscription than their standard equivalents. Appetite for green bonds is mobilising increased capital markets investment to meet climate goals and environmental protection. While there is no universal definition of green investors, there is a clear evidence that the amount of green bonds allocated to investors declaring themselves as green is constantly growing (CBI 2018). The success of these instruments reflects the fact that investors are increasingly conscious of the environmental consequences of the decisions that companies and governments make and are ready to exchange financial performance with the assurance of a more sustainable world.

In our paper we showed that alongside the obvious benefits that green bonds give to the development of sustainable investing, they indirectly contribute to an enhancement of a company’s debt quality and creditworthiness, and thus they may offer stability in times of market volatility, which investors and governments ultimately like.

## Appendix

In this section we search for an approximate solution of the free boundary-value problem:

22 $$\Psi V = 0$$

for the differential operator

23 $$\Psi = \frac{{\sigma^{2} }}{2}Y^{2} \partial_{Y}^{2} + \mu Y\partial_{Y} - r + \frac{{s^{2} }}{2}\partial_{X}^{2} + \rho \sigma sY\partial_{XY}^{2}$$

with the boundary conditions:

24 $$\begin{aligned} & V\left( {X,Y^{*}\left( X \right)} \right) = - Vp\left( {X,Y} \right) = \frac{{g\left( {1 - \tau } \right)}}{r} - \Omega \left( X \right)Y^{*} \left( X \right), \\ & \partial_{X} V\left( {X,Y^{*}\left( X \right)} \right) \, = P_{g} Y^{*} (X), \partial_{Y} V\left( {X,Y^{*}\left( X \right)} \right) = - \Omega (X), \\ & \lim_{Y \to \infty } V(X,Y)/Y < \infty \; {\text{for}}\,{\text{ any}}\;{\text{ X}} > 0, \\ \end{aligned}$$

where $\Omega \left( X \right) = Z_{g} - P_{g} X$ and the notation is as in Sect. 3.

In what follows we construct an approximate solution to (22) which is the homogeneous version of Eq. (3) in Sect. 3. If we make an Ansatz of the form $A\left( X \right)Y^{\beta }$ then A(X) and β should satisfy:

$$\frac{{\sigma^{2} }}{2}\beta \left( {\beta - 1} \right)A\left( X \right) + \mu \beta A\left( X \right) - rA\left( X \right) + \frac{{s^{2} }}{2}A^{{\prime \prime }} \left( X \right) + \rho \sigma sA^{{\prime }} \left( X \right) = 0$$

where the primes denote differentiation with respect to X. Note that there is a multitude of choices for A(X) depending on β, although our choice is restricted by the boundary conditions (24). In view of the peculiar shape of the boundary conditions a reasonable guess is that A(X) is a function of Ω(X), which is what we assume in the sequel. Changing to variables: $y = \Omega \left( X \right)Y$ and *V(X,Y)* = *v(y),* (A1) is written in the form:

$$\psi_{0} v = \varphi v = :f$$

where

$$\psi_{0} = \frac{1}{2}\left[ {\sigma^{2} + \left( {sz} \right)^{2} - \rho \sigma sz} \right]y^{2} \frac{{d^{2} }}{{dy^{2} }} + \left( {\mu - \rho \sigma sz} \right)\frac{d}{dy} - r$$

with $$z = P_{g} /\Omega \left( 1 \right)$$, and

$$\varphi = \left[ {\Omega \left( 1 \right) - \Omega \left( X \right)} \right]\left\{ {\left[ {\frac{\rho \sigma sz}{{\Omega \left( X \right)}} - \frac{{\left( {sz} \right)^{2} \left( {\Omega \left( 1 \right) + \Omega \left( X \right)} \right)}}{{2\left( {\Omega \left( X \right)} \right)^{2} }}} \right]y^{2} \frac{{d^{2} }}{{dy^{2} }} + \frac{\rho \sigma sz}{{\Omega \left( X \right)}}y\frac{d}{dy}} \right\}.$$

We assume that *X* is in a compact neighbourhood of 1 such that $\Omega \left( X \right) \ge c_{0} > 0$, so that our argument holds for X close to 1.

First, we look for a general solution of $\psi_{0} v = 0$ satisfying $lim_{y \to \infty } v\left( y \right)/y < \infty .$

Then *v* is of the form $v_{0} \left( y \right) = A_{0} y^{\beta }$, where β is the negative solution of the algebraic equation associated with the differential equation $\psi_{0} v = 0$, that is,

$$\beta = \left[ {\frac{{\sigma^{2} + (sz)^{2} }}{2} - \mu - \sqrt {\left( {\frac{{\sigma^{2} + (sz)^{2} }}{2} - \mu } \right)^{2} + 2r(\sigma^{2} + (sz)^{2} - 2\rho \sigma sz}) } \right]\left / \right[\sigma^{2} + \left( {sz)^{2} - 2\rho \sigma sz} \right].$$

Define *y** = $$\frac{\beta }{\beta - 1}\frac{{g\left( {1 - \tau } \right)}}{r}$$ and $$A_{0} = \frac{1}{ - \beta }[\frac{\beta }{\beta - 1}\frac{{g\left( {1 - \tau } \right)}}{r}]^{1 - \beta }$$.

Then $v_{0} \left( {y^{*} } \right) = \frac{{g\left( {1 - \tau } \right)}}{r} - y^{*}$ and $v_{0}^{^{\prime}} \left( {y*} \right) = - 1$, which implies that $V_{0} \left( {X,Y} \right) = v_{0} \left( {\Omega \left( X \right)Y} \right)$ satisfies the boundary conditions (A3) with $Y^{*} \left( X \right) = y^{*} \Omega \left( X \right).$

Note that $f\left( y \right) = v_{0} \left( y \right)O\left( {X - 1} \right)$. If we define $v\left( y \right) = v_{0} \left( y \right) + \frac{2}{{\Sigma_{ - }^{2} \left( {\beta - \beta ^{\prime}} \right)}}\mathop \smallint \limits_{y*}^{y} \frac{f\left( t \right)}{{t^{\beta + 1} }}dt$, where β’ is the positive solution of the algebraic equation associated with $\psi_{0} v = 0$ and $\Sigma_{ - }^{2} = \sigma^{2} + \left( {sz} \right)^{2} - 2\rho \sigma sz$, then $\psi v = 0$ up to an error which is $O\left( {\left( {X - 1} \right)^{2} } \right)$. Moreover, *v* satisfies the same pasting condition as $v_{0}$ and the same smooth-pasting condition up to $O\left( {X - 1} \right).$ Note that $v\left( y \right) = v_{0} \left( y \right)\left[ {1 + O\left( {X - 1} \right)} \right]$, so $V_{0} \left( {X,Y} \right)$ may serve as a reasonable approximate solution provided that *X* is sufficiently close to 1, and the error is $O\left( {X - 1} \right)$.

## Footnotes

1 Strong demand for green bonds continued during 2020, although the pandemic triggered a shift in demand for social bonds slowing down green bonds in favour of bonds linked to specific coronavirus measures. On 15 Dec. 2020, CBI announced that in Q4 green bonds reached their most substantial milestone, surpassing 1tr USD in cumulative issuance since market inception in 2007.

2 The Green Bond Principles provide that issuers should report at least annually on project details performance updates, and, where feasible, on the environmental impact assessment.

3 A large academic literature and practitioner debate has examined the relationship between corporate social responsibility, corporate financial performance and returns to responsible investing. Amel-Zadeh (2018) reviews a comprehensive survey of this literature and discusses methodological issues and difficulties with causal inference of most of these empirical studies.

4 According to Harrison et al. (2020), approximately 50% of green bonds are allocated to investors declaring themselves as green responsible.

5 In the ‘Risk Factors’ section of US firm’s 10-K filing with the SEC, uncertainty about the impact of climate policy is often listed among the key risks. The Network for Greening the Financial System, established in 2017, acknowledges climate-related financial risks as relevant risk factors which have an impact in asset valuation and climate risks have started being integrated in macro-prudential frameworks by several central banks as well.

6 Harrison et al. (2020) report that most respondents in their Survey are aware that investors want more information on the use of proceeds and post issuance transparency, and 85% of investors would either sell or be inclined to sell a green bond if post issuance reporting is poor.

7 A real-world situation is offered by the car industry, where a set of penalties have been established for those firms that do not achieve a scheduled environmental standard. However, other forms of reduction of the firm earnings are encompassed in this framework, e.g. due to reputational issues (Orlitzky et al. 2003; Bènabou and Tirole 2010; Heal 2005).

8 See the Climate Bond Initiative Report (2018).

9 This is a realistic assumption, as most green bonds exhibit an investment grading rating.
